# Semi-Automatic Algorithms for Estimation and Tracking of AP-Diameter of the IVC in Ultrasound Images

**DOI:** 10.3390/jimaging5010012

**Published:** 2019-01-09

**Authors:** Ebrahim Karami, Mohamed S. Shehata, Andrew Smith

**Affiliations:** 1 Department of Engineering and Applied Sciences, Memorial University, St. John’s, NL A1B 3X5, Canada; 2 Faculty of Medicine, Memorial University, St. John’s, NL A1B 3V6, Canada

**Keywords:** inferior vena cava (IVC), ultrasound imaging, anterior posterior (AP) diameter, active ellipse, active rectangle, volume status

## Abstract

Acutely ill patients presenting with conditions such as sepsis, trauma, and congestive heart failure require judicious resuscitation in order to achieve and maintain optimal circulating blood volume. Increasingly, emergency and critical care physicians are using portable ultrasound to approximate the temporal changes of the anterior–posterior (AP)-diameter of the inferior vena cava (IVC) in order to guide fluid administration or removal. This paper proposes semi-automatic active ellipse and rectangle algorithms capable of improved and quantified measurement of the AP-diameter. The proposed algorithms are compared to manual measurement and a previously published active circle model. Results demonstrate that the rectangle model outperforms both active circle and ellipse irrespective of IVC shape and closely approximates tedious expert assessment.

## 1. Introduction

Acutely ill patients presenting with conditions such as sepsis, trauma, and congestive heart failure require judicious resuscitation in order to achieve and maintain optimal circulating blood volume while avoiding increased morbidity and mortality [1,2,3,4]. Increasingly, emergency and critical care physicians visually approximate the AP-diameter of the IVC using portable ultrasound in order to guide fluid management on these patients [5,6,7]. Unfortunately, ultrasound image quality is highly operator dependant, images contain considerable noise, and image artifacts frequently impede accurate manual or computerized segmentation [8,9].

Speckle noise present in ultrasound imagery is theoretically considered to be a Rayleigh distributed multiplicative noise [10] as the envelop of the ultrasound wave reflected from each tissue has a Rayleigh distribution. Hence, Rayleigh mixture models have been proposed as a solution for ultrasound image segmentation [11,12]. However, it has been shown that due to the scattering population and signal processing, the speckle distribution deviates from Rayleigh [13]. In [14], authors proposed a fast algorithm based on optical flow for tracking of the speckles in ultrasound images. This approach fails when the speckle structure is rapidly deformed.

Active contours (ACs) are widely used for segmentation of ultrasound images [15,16,17,18,19,20]. ACs convert the problem of image segmentation into a minimization of an energy functional with their performance frequently dependent on a manually defined initialization contour. In order to avoid local minima, the initiating contour needs to be as close as possible to the actual contour. ACs can be combined with other segmentation algorithms as a coarse-to-fine strategy to reduce the impact of the initial contour on segmentation error [21,22]. Researchers have addressed the challenge of IVC segmentation using this strategy by using template matching method as the coarse segmentation and AC as the fine-tuning (TMAC) [23]. Unfortunately, this approach fails when the IVC undergoes large frame-to-frame variations commonly present on portable machines with lower frame rates (e.g., 30 frames-per-second). Additionally, ACs continue to perform poorly in the context of fuzzy or unclear boundaries as is commonly the case for the IVC.

Given that the cross-section of the IVC is largely convex, the IVC contour can be represented in polar coordinates and consequently, polar active contours appear as a promising solution for IVC segmentation [24]. In [25], a polar AC model based on the third centralized moment (M3) was proposed for segmentation of IVC images. Unfortunately, M3 algorithm roughly estimates the cross-sectional area (CSA) of the IVC and fails with poor quality images. In [26], adaptive polar AC algorithms were proposed for segmentation of ultrasound images, where the parameters of the AC models are locally and temporally adapted as frame by frame basis. This approach fails with poor quality IVC images [27].

Accurate segmentation algorithms such as the one proposed in this manuscript have the potential to expand understanding into non-invasive volume status monitoring. Clinical research has demonstrated that the true angle of collapse is actually 25 degrees off vertical rather than the simple AP-diameter [28].

In [26], the off-axis collapse was appropriately modeled using the diameter of a circle fitted inside the IVC with good results; however, tools capable of measuring the CSA, AP-diameter, and off-axis collapse are needed. In this paper, we propose two algorithms based on ellipse and rectangle models. The height of a thin rectangle fitted inside the IVC can efficiently model its clinically measured AP-diameter. We also develop another algorithm based on ellipse fitting just for comparison purpose.

The remainder of this paper is organized as follows: Section 2 discusses the background and related work. The proposed active rectangle and active ellipse algorithms are presented in Section 3 while experimental results are in Section 4, the results are discussed in Section 5 and the paper is concluded in Section 6.

## 2. Background and Related Work

In [26], authors showed that the AP-diameter of the IVC can be accurately modeled with the diameter of a circle fitted inside the IVC. The active circle algorithm proposed in [26] is based on the following evolution functional:

(1) E=α(u−v)(2I−u−v),

where *u* and *v* are the mean of the intensities for the pixels inside and outside the contour, respectively, and *I* is the intensity of the pixels on the contour. This functional is used to evolve the parameters of the circle, i.e., *R* as the circle radius and $\left(x_{c},y_{c}\right)$ as its center coordinates. For this, the circle is sampled at *K* points with polar angles $\theta_{k}=\frac{2k\pi }{N}$, k=0,1,…,K−1, where the normal vector and the Cartesian coordinates corresponding to the *k*th sampled point are denoted as

(2) $${\vec{n}}_{k}={\left[\cos\left(\theta_{k}\right),\sin\left(\theta_{k}\right)\right]}^{T},$$

and

(3) $${\left[x_{k},y_{k}\right]}^{T}={\left[x_{c},y_{c}\right]}^{T}+R{\vec{n}}_{k},$$

respectively. The evolutional functional generates forces $f_{k}$ along the normal vectors as

(4) $$f_{k}=\alpha \left(u-v\right)\left(2I_{k}-u-v\right).$$

These forces move the contour points to their new positions as

(5) $$\left[{\tilde{x}}_{k},{\tilde{x}}_{k}\right]={\left[x_{c},y_{c}\right]}^{T}+\left(R+f_{k}\right){\vec{n}}_{k},$$

where $f_{k}$ is the value of the evolution functional at *k*th contour point. It is shown that the average evolution functional approaches zero when the contour points are on the IVC boundary. In active circle algorithm, the center of the circle is evolved as

(6) $$\left[{\tilde{x}}_{c},{\tilde{y}}_{c}\right]=\left[x_{c},y_{c}\right]+\frac{1}{K}\sum_{k=0}^{K-1}f_{k}{\vec{n}}_{k},$$

where $\left[x_{c},y_{c}\right]$ and $\left[{\tilde{x}}_{c},{\tilde{y}}_{c}\right]$ are the center coordinates of the circle before and after evolution, respectively, and $f_{k}$ is the evolution force for the *k*th contour points obtained from 1, and ${\vec{n}}_{k}$ is its corresponding normal vector that can be obtained as

(7) $${\vec{n}}_{k}={\left[\cos\left(\theta_{k}\right),\sin\left(\theta_{k}\right)\right]}^{T},$$

Equation (6) indicates that the circle center point is simply translated by the average of the force vectors $f_{k}{\vec{n}}_{k}$. The center of the circle is evolved as

(8) $$\tilde{R}=R+\frac{1}{K}\sum_{k=0}^{K-1}f_{k}.$$

where *R* and $\tilde{R}$ is the radius of the circle before and after evolution, respectively. This indicates that the circle radius is evolved with the average of the force magnitudes $f_{k}$.

## 3. Proposed Algorithms

In this section, we develop active ellipse and rectangle models based on the evolution functional proposed in [26].

### 3.1. Active Ellipse Model

In [26], authors showed that the active circle algorithm estimates the IVC AP-diameter much more accurate than the Star–Kalman algorithm in [29] which is based on ellipse fitting, but does this indicate that a circular model can estimate the AP-diameter more accurate than an elliptical model? To answer this question, we develop an active ellipse algorithm based on the same evolutional functional as the one employed in the active circle algorithm.

In general case, during the evolution, the *k*th contour point is evolved as

(9) $$\left[{\tilde{x}}_{k},{\tilde{x}}_{k}\right]={\left[x_{k},y_{k}\right]}^{T}+f_{k}{\vec{n}}_{k}.$$

The next step is to evolve the ellipse parameters using the evolved contour points. Unlike the circular model, the evolved ellipse parameters are not linearly related to evolved contour points. This non-linearity may result in convergence to local minima. To avoid this, at each iteration, we fit a new ellipse using the following conic equation. Note that since the algorithm still operates iteratively, the ellipse parameters are gradually evolving and hence, we call this algorithm active ellipse algorithm.

(10) $$ax^{2}+bxy+cy^{2}+dx+ey=1,$$

where *x* and *y* are the coordinates of the points on the conic, *a*, *b*, *c*, *d*, and *e* are the conic parameters. Note that with an elliptical model, the values of *a* and *b* must be positive. With *K* points with coordinates $\left[{\tilde{x}}_{k},{\tilde{x}}_{k}\right]$, the best ellipse is fitted by minimizing the following cost function:

(11) $$C=\sum_{k=1}^{K}{\left(a{\tilde{x}}_{k}^{2}+b{\tilde{x}}_{k}{\tilde{y}}_{k}+c{\tilde{y}}_{k}^{2}+d{\tilde{x}}_{k}+e{\tilde{y}}_{k}-1\right)}^{2}.$$

Equation (11) can be rewritten in matrix form

(12) $$C\left(A\right)=A^{T}X^{T}XA+21_{K}^{T}XA+K,$$

where the vector of conic parameters defined as $A={\left[a,b,c,d,e\right]}^{T}$, *X* is a matrix with $\left[{\tilde{x}}_{k}^{2},{\tilde{x}}_{k},{\tilde{y}}_{k},{\tilde{y}}_{k}^{2},{\tilde{x}}_{k},{\tilde{y}}_{k}\right]$ as its *k*th row, $1_{K}$ is K×1 all-one vector, and superscript *T* is the transpose operator. After setting the gradient C(A) to zero, the vector *A* is obtained as

(13) $$\hat{A}=-1_{K}^{T}X{\left(X^{T}X\right)}^{-1}.$$

**Figure 1:**
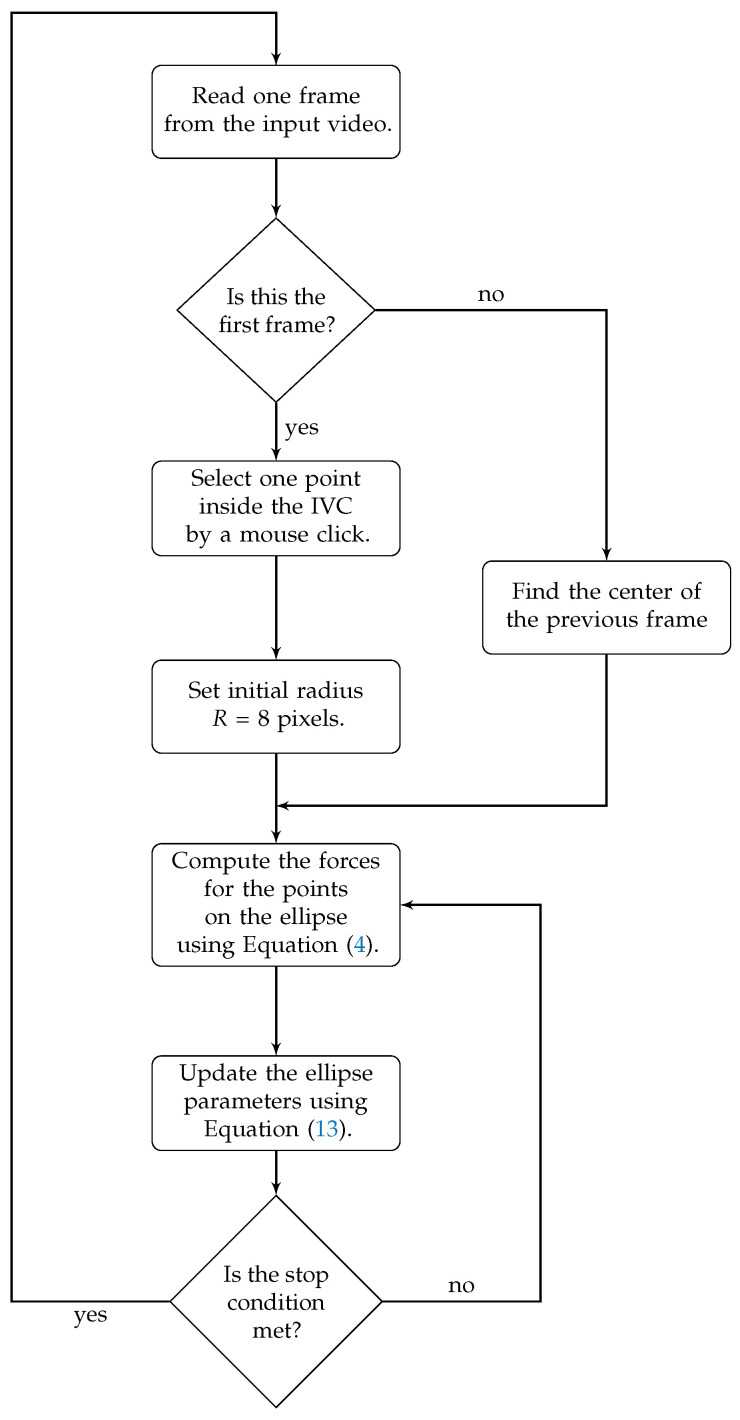
Flowchart for the proposed active ellipse algorithm for estimation and tracking of the IVC AP-diameter from ultrasound videos.

Figure 1 presents the flowchart for the proposed active ellipse algorithm. As shown in the flowchart, only for the first frame, an operator needs to manually select a point inside the IVC and the rest of the algorithm works automatic. For each frame, the ellipse parameters are iteratively updated using Equations (4) and (13) until a convergence is reached. We assume that the algorithm has reached the convergence when the maximum change in the elements of the *A* is less than $10^{-4}$, i.e., when $max(|{\hat{A}}_{n}-{\hat{A}}_{n-1}\left|\right)<10^{-4}$, where max is the element-wise maximum, and ${\hat{A}}_{n}$ is the vector *A* estimated at *n*th iteration. After convergence, the algorithm proceeds with the next frame.

**Figure 2:**
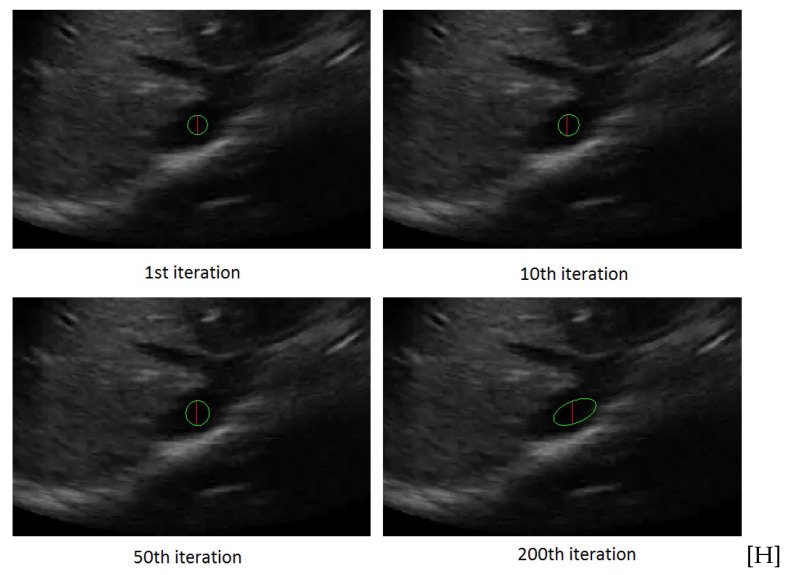
The rectangle evolution versus number of iterations.

Figure 2 shows the ellipse evolution versus the number of iterations for a sample IVC frame.

### 3.2. Active Rectangle Model

The intuition to use a rectangular model is that the AP-diameter is clinically defined as the largest vertical diameter of the IVC contour which may practically deviate from the actual diameter of an circle or even an ellipse. The AP-diameter can be modeled as the height of a vertical thin rectangle. As a starting point, we assume that the fitted rectangle has a fix width *w* = 3 pixels and the only parameters that have to be modified are the center and the height of the rectangle. With the forces defined as Equation (4), either of the upper and lower sides of the rectangle move with the average of the forces applied on that side. Hence, the center of the rectangle is shifted as

(14) $${\tilde{x}}_{c}=x_{c}+\frac{1}{K_{l}}\sum p\in P_{l}f_{p}-\frac{1}{K_{r}}\sum p\in P_{r}f_{p},$$

(15) $${\tilde{y}}_{c}=y_{c}+\frac{1}{K_{u}}\sum p\in P_{u}f_{p}-\frac{1}{K_{b}}\sum p\in P_{b}f_{p},$$

where $P_{l}$, $P_{r}$, $P_{u}$, and $P_{b}$ are the subsets of the contour points on the left, right, upper, and lower sides of the rectangle, respectively, and $K_{l}$, $K_{r}$, $K_{u}$, and $K_{b}$ are the number of points in each of these sets. Similarly, the height of the rectangle is evolved as

(16) $$\tilde{h}=h+\frac{1}{K_{u}}\sum p\in P_{u}f_{p}+\frac{1}{K_{b}}\sum p\in P_{b}f_{p}.$$

**Figure 3:**
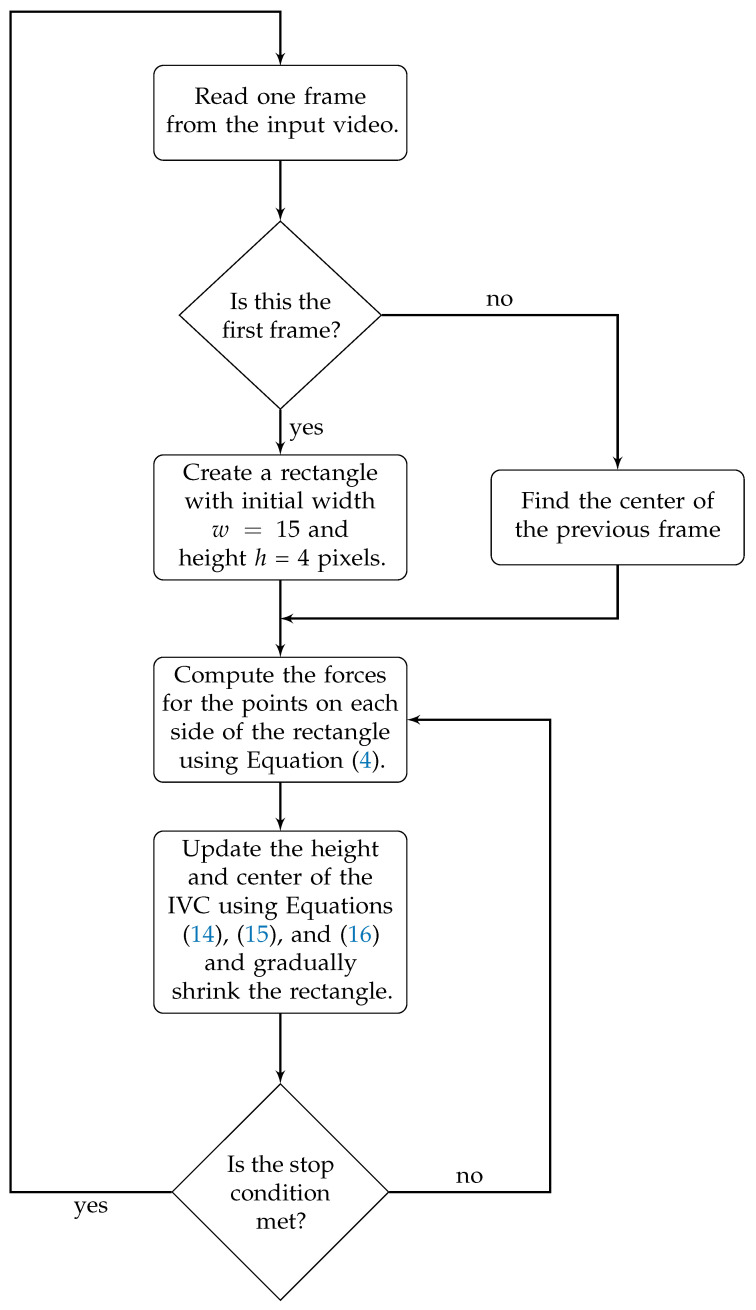
Flowchart for the proposed active rectangle algorithm for estimation and tracking of the IVC AP-diameter from ultrasound videos.

Although a thin rectangle accurately models the clinically measured AP-diameter, it might be lost if parts of the IVC boundaries are missing as the IVC edges are not detected and hence, the algorithm may diverge. To combat this problem, we modify the active rectangle algorithm by starting with a rectangle with a much larger width as *w* = 15 pixels. This rectangle is gradually narrowed to its final width, i.e., *w* = 3 pixels which is narrow enough to model the AP-diameter. The active rectangle algorithm is summarized as the flowchart in Figure 3. As shown in Figure 3, similar to the active circle and ellipse algorithms, an operator has to manually select a point inside the IVC and the rest of the algorithm is automatic and does not need further manual intervene. The active rectangle algorithm converges much faster than active circle and ellipse algorithms as the results show that in all cases, no more than $N_{i}$ = 200 iterations are required to reach a convergence, while with other two algorithms, typically up to $N_{i}$ = 5000 iterations are required to reach a convergence. Therefore, there is no need to set a stop condition for the active rectangle algorithm.

**Figure 4:**
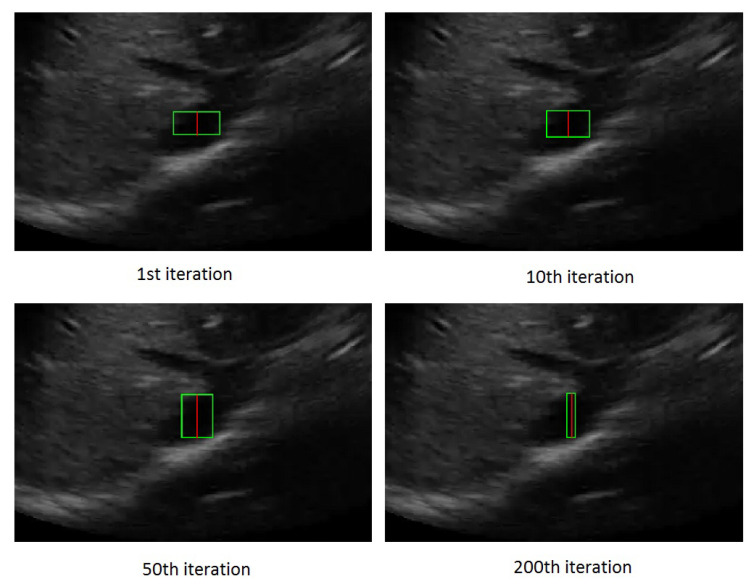
The rectangle evolution versus number of iterations.

Figure 4 shows the rectangle evolution versus number of iterations for the IVC image as in Figure 2. By comparison of Figure 2 and Figure 4, one can see the active rectangle algorithm not only converges faster but also more accurately estimates the AP-diameter than the active ellipse algorithm.

## 4. Results

**Figure 5:**
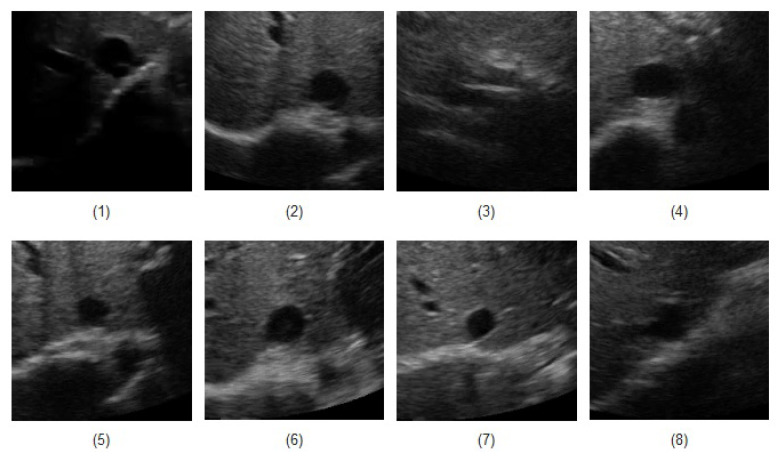
The first frame of all eight IVC videos.

Ultrasound videos from eight healthy subjects (The data was actually collected from twenty subjects, but only for eight cases, the manual measurement seemed reliable to be used as the ground truth). were collected with the IVC imaged in the transverse plane using a portable ultrasound (M-Turbo, Sonosite-FujiFilm) and a phased-array probe (1–5 MHz). Each video has a frame rate of 30 fps, scan depth of 19 cm, and a duration of 15 s (450 frames/clip). Figure 5 depicts the first frame of all eight subjects. In Figure 5, one can see that an IVC image can have different shapes and qualities. For instance, in the clip no. (1), although part of the image is shadowed, but the IVC edges are almost visible. The IVC videos for the clips nos. (3) and (8) show the lowest quality as the IVC is almost collapsed in the former one and it vanishes after the initial frames in the latter one (this is not seen in this image as it is only the first frame of the video).

### Tracking Performance

**Figure 6:**
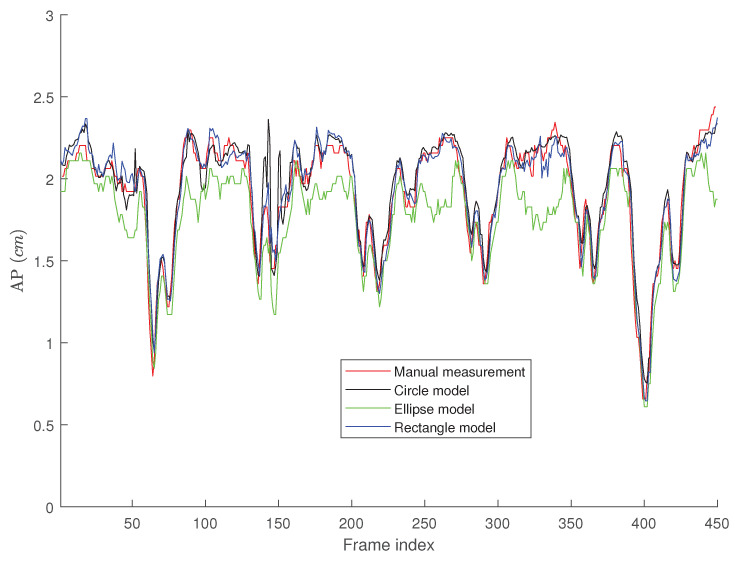
AP-diameter for the first video depicted in Figure 5, as measured by the manual measurement (red line), active circle algorithm (black line), active ellipse algorithm (green line) and active rectangle algorithm (blue line).

**Figure 7:**
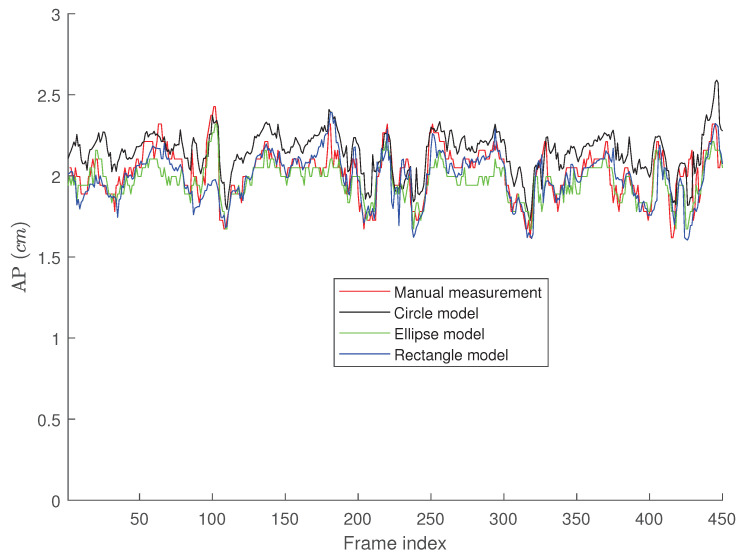
AP-diameter for the second video depicted in Figure 5, as measured by the manual measurement (red line), active circle algorithm (black line), active ellipse algorithm (green line) and active rectangle algorithm (blue line).

**Figure 8:**
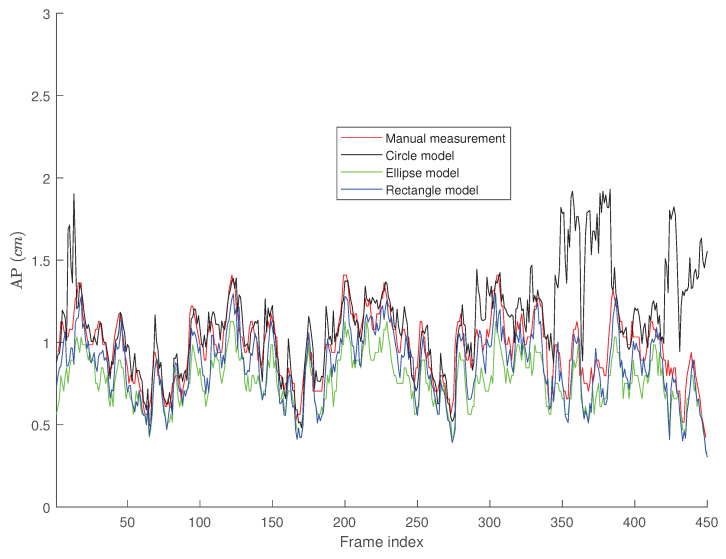
AP-diameter for the third sample video depicted in Figure 5, as measured by the manual measurement (red line), active circle algorithm (black line), active ellipse algorithm (green line) and active rectangle algorithm (blue line).

Figure 6, Figure 7 and Figure 8 present the AP-diameter manually measured by Dr. Andrew Smith as a point-of-care ultrasound expert and the ones that are semi-automatically estimated by the three shape-based algorithms for the first three sample videos depicted as subjects (1)–(3) in Figure 5. From Figure 6, one can see that with the first IVC clip which has a good quality, both active circle and active rectangle algorithms efficiently track the manual measurement, although the active ellipse algorithm roughly tracks the manual measurement and performs poorer than the other two methods.

Figure 7 presents the tracking results for the second clip where one can easily see that all three algorithms perform less accurate than the case in Figure 6. This is mainly due to the fact that although the second clip seems to have a better quality than the first one, it has a more fuzzy contour, making the algorithms less accurate than the first clip. Similar to the first clip, we can see that the active rectangle algorithms performs better than the other two methods.

Figure 8 presents the tracking results for the third video depicted in Figure 5 where the IVC is almost collapsed and therefore, a smaller AP-diameter is expected. In this case, the active circle algorithm loses the tracking after 337 frames, while both active ellipse and active rectangle algorithms efficiently track the result obtained from the manual measurement.

**Figure 9:**
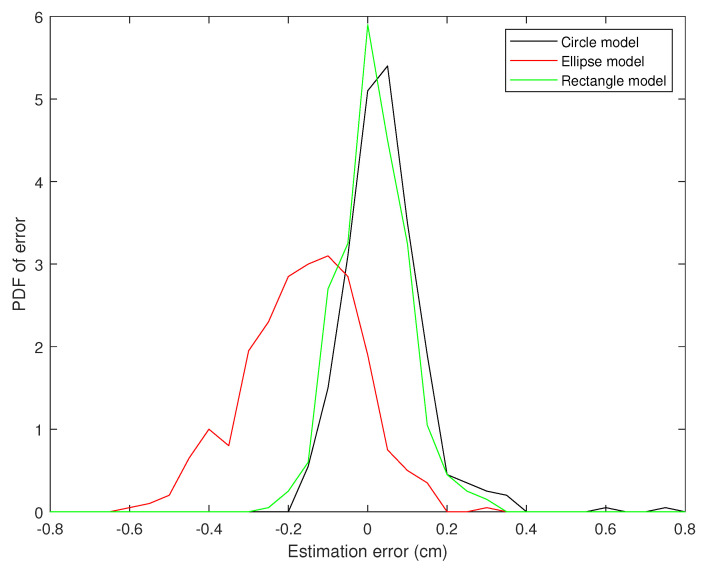
PDF of the AP-diameter estimation error w.r.t. manual measurement for the first sample video depicted in Figure 5, as measured by active circle algorithm (black line), active ellipse algorithm (red line) and active rectangle algorithm (green line).

**Figure 10:**
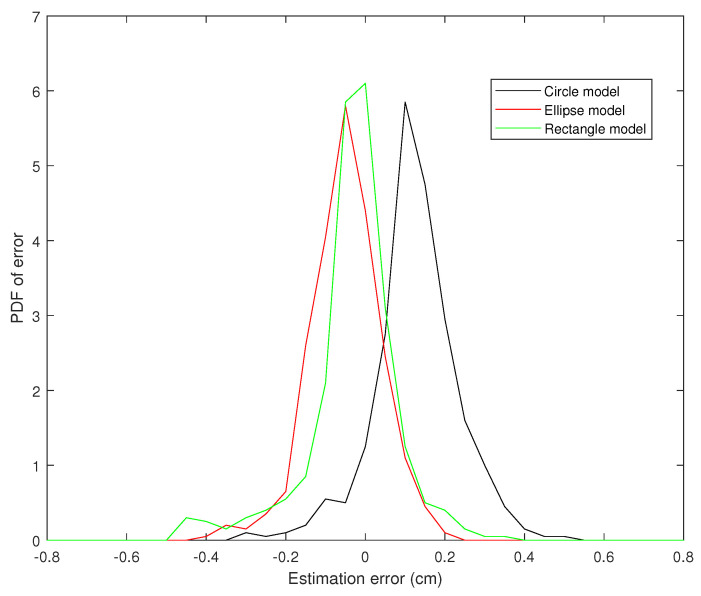
PDF of the AP-diameter estimation error w.r.t. manual measurement for the second sample video depicted in Figure 5, as measured by active circle algorithm (black line), active ellipse algorithm (red line) and active rectangle algorithm (green line).

**Figure 11:**
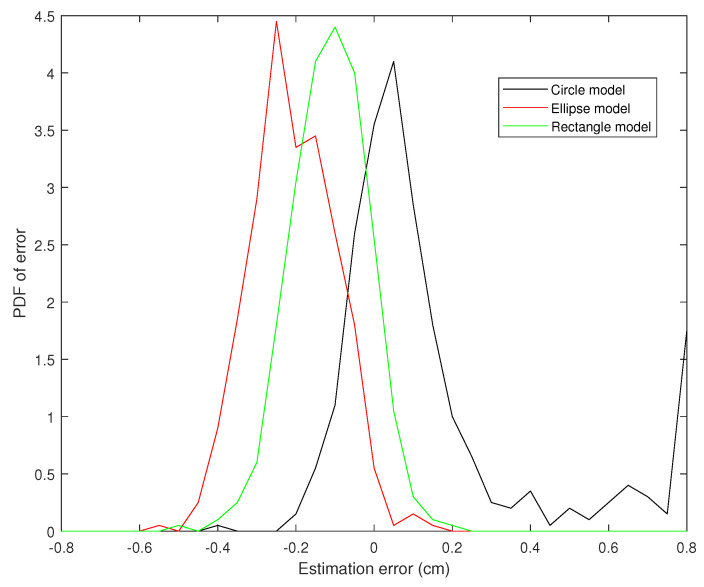
PDF of the AP-diameter estimation error w.r.t. manual measurement for the third sample video depicted in Figure 5, as measured by active circle algorithm (black line), active ellipse algorithm (red line) and active rectangle algorithm (green line).

Figure 9, Figure 10 and Figure 11 present the probability distribution function (PDF) of the AP-diameter estimation error for the three videos depicted as subjects (1)–(3) in Figure 5. Here, error is defined as the difference between the AP-diameter estimated by each of the three shape-based algorithms and the one measured by the expert. From Figure 9, Figure 10 and Figure 11, one can see that for all three investigated scenarios, the active circle algorithm provides a biased measurement. This confirms the similar result reported in [26]. Furthermore, for all three cases the PDF of the error obtained from the active rectangle algorithm is more concentrated around zero than the other two algorithms, indicating the best performance among the three shape-based algorithms.

Figure 6, Figure 7, Figure 8, Figure 9, Figure 10 and Figure 11 show that in all three investigated clips, the active rectangle algorithm outperforms the other two methods. To have a better insight about the results, we present numerical results in Table 1 and Table 2. Table 1 presents the room mean square (RMS) of the AP-diameter estimation error for the three shape-based algorithms for the all eight clips depicted in Figure 5. Note that the error is defined as the difference between the AP-diameter estimated by each of the algorithms and the one manually measured by the expert. Except with subject no. (8), where manual segmentation is reliable for only the first 150 frames (see [26]), for the other seven subjects, the RMS of error is calculated over all 450 frames. From this table, one can see that in all eight investigated cases, the active rectangle algorithm outperforms the other two methods, while in five out of the eight cases, the active circle algorithm performs more accurate than the active ellipse algorithm.

Table 2 presents the maximum absolute value of error obtained for the three algorithms for all eight clips depicted in Figure 5. This tables confirms the results obtained in Table 1, i.e., the active rectangle algorithms always outperform the other two methods.

Table 3 present the correlation between the AP-diameter estimated by each of the three shape-based algorithms and the one measured by the expert. This table confirms the results obtained in Table 1 and Table 2 as in all eight cases, the proposed active rectangle algorithm outperforms the other two algorithms and provides the highest correlation with the manual measurement.

Table 4 present the average position error for each of the three shape-based algorithm versus the one measured by the expert. In this table, the distance position error is defined as the Euclidean distance (in cm) between the center of the fitted shape (circle, ellipse, or rectangle) and the center of the AP-line manually located by the expert. This also confirms the previous results as in all eight investigated cases the center of the fitted rectangle is closer to the manual measurement than the centers of the fitted circle and ellipse.

## 5. Discussion

### 5.1. The Performance of the Proposed Algorithms

As it was described earlier in the Results section, for all eight investigated clips, the active rectangle algorithm performs closer to the manual measurement than the other two methods. This is due to the fact the AP-diameter is clinically defined as the largest vertical diameter inside the IVC. The active circle algorithm finds the largest circle inside the IVC and assumes that the diameter of this circle can efficiently model the AP-diameter. This is technically correct if the IVC is horizontally aligned, but based on the angle of the ultrasound prob, the IVC in the ultrasound clip can be rotated along the horizontal axis and this makes the results somewhat different from the clinically measured AP-diameter.

In five out of the eight cases, the active circle algorithm performed better than the active ellipse algorithm. This is mainly due to the fact that a circle has less degree of freedom than an ellipse and therefore, circle evolution can be performed more accurate than ellipse evolution. Furthermore, from Figure 5, one can see that in most cases, the IVC does not appear to have an elliptical shape making the ellipse fitting process inaccurate.

### 5.2. Complexity Comparison

In this section, we analyze the computational complexity of the proposed algorithms in terms of the number of floating points operations (flops) required to estimate the AP-diameter for each frame. Assume $N_{i}$ as the maximum number of iterations required to reach a convergence, *K* as the number of contour points, and $A_{max}$ as the maximum number of pixels inside the fitted shape. The maximum required flops per frame for active circle algorithm is $N_{flops}^{cir}\approx \left(2A_{max}+3K\right)N_{i}$. From Equations (4), (13)–(16), one can see that the maximum required flops per frame for active ellipse and rectangle algorithms are $N_{flops}^{ell}\approx \left(2A_{max}+5K\right)N_{i}$ and $N_{flops}^{rec}\approx \left(2A_{max}+2K\right)N_{i}$, respectively. Note that the rectangular model converges much faster than circular and elliptical models as the active circle and ellipse algorithms need up to $N_{i}=5000$ iterations to reach a convergence, while the active rectangle algorithm converges in no more than $N_{i}=200$ iterations.

## 6. Conclusions

In this paper, two novel algorithms based on elliptical and rectangular models were proposed for semi-automatic estimation of AP-diameter of the IVC in ultrasound videos. The proposed algorithms were compared with the active circle algorithm and was shown that although IVC usually has an elliptical CSA, both circular and rectangular models provide a more accurate AP-diameter measurement, while the rectangular model outperforms the other two models. This is due that fact that the AP-diameter is clinically measured as the maximum vertical diameter of the IVC which can be modeled better as the vertical side of a rectangle than the diameter of a circle or even the largest vertical diameter of an ellipse.

## Figures and Tables

**Table 1:** RMS of the AP-diameter estimation error.

	Subject No.	(1)	(2)	(3)	(4)	(5)	(6)	(7)	(8)	Ave.
Method										
**Circle model**		0.10	0.16	0.16	0.25	0.25	0.10	0.11	0.26	0.17
**Ellipse model**		0.21	0.19	0.14	0.26	0.18	0.11	0.11	0.20	0.35
**Rectangle model**		0.08	0.11	0.12	0.23	0.14	0.10	0.10	0.18	0.12

**Table 2:** Maximum absolute value of AP-diameter estimation error.

	Subject No.	(1)	(2)	(3)	(4)	(5)	(6)	(7)	(8)
Method									
**Circle model**		0.06	0.29	0.48	0.57	0.75	0.41	0.44	0.43
**Ellipse model**		0.11	0.32	0.35	0.59	0.48	0.54	0.47	0.38
**Rectangle model**		0.05	0.18	0.19	0.42	0.28	0.37	0.39	0.29

**Table 3:** Correlation between the AP-diameters estimated by the three shape-based algorithms and manual measurement.

	Subject No.	(1)	(2)	(3)	(4)	(5)	(6)	(7)	(8)
Method									
**Circle model**		0.9987	0.9987	0.9652	0.7971	0.9985	0.9986	0.9988	0.9958
**Ellipse model**		0.9974	0.9985	0.9945	0.9981	0.9986	0.9985	0.9991	0.9850
**Rectangle model**		0.9993	0.9994	0.9949	0.9991	0.9992	0.9991	0.9994	0.9985

**Table 4:** The average position error for the three shape-based algorithms w.r.t. the manual measurement.

	Subject No.	(1)	(2)	(3)	(4)	(5)	(6)	(7)	(8)	Ave.
Method										
**Circle model**		0.41	0.39	0.63	0.65	0.36	0.49	0.55	0.64	0.51
**Ellipse model**		0.42	0.43	0.31	0.47	0.44	0.49	0.54	0.56	0.45
**Rectangle model**		0.37	0.33	0.27	0.28	0.29	0.47	0.54	0.55	0.39

## Abbreviations

The following abbreviations are used in this manuscript:

AP	Anterior–posterior
IVC	Inferior vena cava
CSA	Cross sectional area.
